# Grazing in the dark: A behavioural adjustment in a population of the black sea urchin *Arbacia lixula*

**DOI:** 10.1002/ece3.10428

**Published:** 2023-08-31

**Authors:** Simone Mariani, Susana Pinedo, Esther Jordana, Maria Elena Cefalì, Xavier Torras, Marina Bagur Bendito, Jana Verdura, Enric Ballesteros

**Affiliations:** ^1^ Centre d'Estudis Avançats de Blanes, Consejo Superior de Investigaciones Científicas (CEAB‐CSIC) Blanes Spain; ^2^ Estació d'Investigació Jaume Ferrer Instituto Español de Oceanografía‐CSIC Maó Spain; ^3^ Observatori Socioambiental de Menorca Institut Menorquí d'Estudis Maó Spain; ^4^ CNRS, UMR, 7035 ECOSEAS Université Côte d'Azure Nice France

**Keywords:** antipredator response, Mediterranean Sea, overgrazing, sea urchin barrens

## Abstract

In Mediterranean rocky shores, the black sea urchin *Arbacia lixula* is often associated with communities dominated by encrusting corallines, devoid of fleshy algae. While it is commonly known as a diurnal herbivore, this species also migrates at night from hidden to more exposed habitats. Here, we provide the first experimental evidence of an adjustment to a predominant nocturnal behaviour in a population of *A. lixula*. Sea urchin densities changed from nearly zero during daytime to more than 16 urchins m^−2^ at night in treatment plots where the sea urchins were removed. We suggest that the observed behaviour was triggered by our experimental manipulations and was a response to the presence of dead conspecifics and small predatory fishes attracted by the urchin culling. Further research is needed to assess whether our findings can be generalised to the behaviour of *A. lixula* in areas where sea urchins are under strong pressure from diurnal predators. In these cases, it is important to perform sea urchin density counts at night to avoid misleading assessments about the herbivore pressure in a littoral area.

## Graphical abstract

Here, we provide the first experimental evidence of an adjustment to a predominant nocturnal behaviour in a population of *Arbacia lixula*. We suggest that the observed behaviour was triggered by our experimental manipulations and was a response to the presence of dead conspecifics and small predator fishes attracted by the urchin culling. It is important to perform sea urchin density counts at night to avoid misleading assessments about the herbivore pressure in a littoral area.

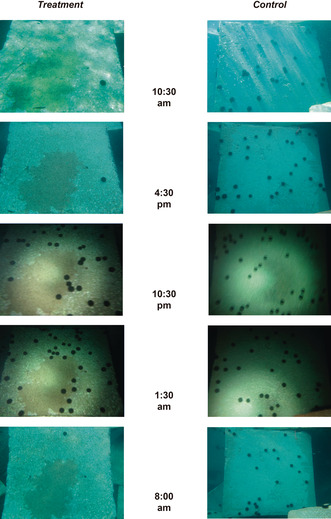

## 1 INTRODUCTION

In Mediterranean rocky shores, the purple sea urchin *Paracentrotus lividus* and the black sea urchin *Arbacia lixula*, often co‐occur in sea urchin barrens devoid of fleshy, frondose algae (herein, FA; Pinna et al., 2020). *A. lixula* has clear warm‐water affinities compared to *P. lividus* and its frequency has been increasing in the last decades, even in the coldest regions of the Mediterranean Sea (Carreras et al., 2021). There is very little information about the behaviours of adult *A. lixula* in the literature, especially as to whether the species is nocturnal or diurnal, or both. One of the few instances is the study of Kempf (1962), who described the night‐time migration of *A. lixula* from hidden to well‐illuminated habitats on artificial boulders in the French coast of Marseille, in the NW Mediterranean Sea. Because of its feeding behaviour (Agnetta et al., 2015), *A. lixula* is particularly fit to maintain barrens dominated by encrusting corallines (herein, EC; Agnetta et al., 2015; Bonaviri et al., 2011). The removal of these herbivores from a habitat may enhance the abundance of FA compared to EC (Bonaviri et al., 2011; Guarnieri et al., 2020), especially in high‐nutrient regions (Boada et al., 2017). Nonetheless, areas in which both EC and FA co‐occur are widespread in many Mediterranean Sea shallow rocky shores. As a matter of fact, the dominance of one community over the other is not regulated by grazing only but by a suite of processes such as nutrient availability, storm regimes, water pollution and propagule availability, whose causal roles are not always easy to assess (Gianguzza et al., 2011).

**FIGURE 1:**
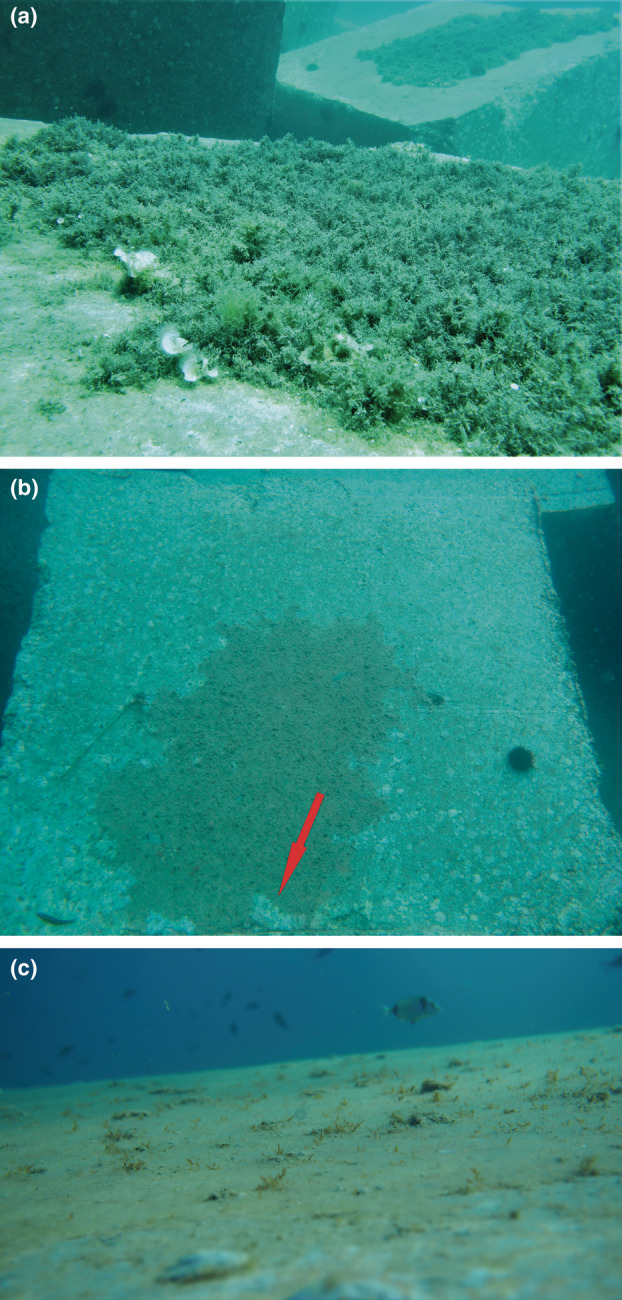
(a) The upper side of a boulder from the study site. Portions of a barren and a community of fleshy, frondose algae are shown. (b) A characteristic feeding trail (red arrow) into the epilithic algal matrix of a treatment plot. (c) Detail of the epilithic algal matrix developed over the upper sides of the boulders in the treatment plots.

**FIGURE 2:**
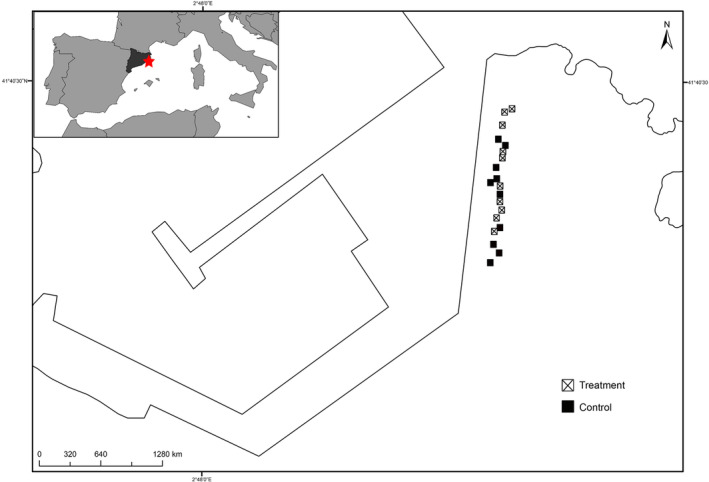
Map of the Blanes breakwater showing the boulders and the experimental set up.

In 2015, our research group had a chance to test if the removal of sea urchins could trigger the growth of FA in sublittoral habitats where both these algae and EC co‐occurred (Figure 1a). Both evidence from aerial images and authors' observations in the field showed that, just after completion of the new breakwater of the Blanes harbour (see Figure 2) in 2012, the underwater portion of the cubic boulders that protect the dock started to become overgrown by FA. In about 1 year, though, the sea urchins *A*. *lixula* and, to a lesser extent, *P. lividus* (see Section 3) colonised the boulders at the same time the FA disappeared leaving in place only barrens with EC (mainly *Lithophyllum incrustans*). In an attempt to achieve the main objective of our study, we eventually witnessed the adjustment to an almost complete nocturnal grazing in the population of *A. lixula* studied.

Here, we describe the results of an experiment carried out to ascertain why, after 4 months of sea urchin removal and in spite of the apparent lack of sea urchins, grazing continued at the treatment plots. We provide evidence for a possible adjustment in the behaviour of *A. lixula* triggered by our experimental manipulations.

## 2 MATERIALS AND METHODS

At the end of July 2015, we haphazardly selected 20 boulders at around 1–3 m depth along the dock of the Blanes harbour (NW Mediterranean), which were devoid of FA. We selected 10 concrete boulders as replicates for the sea urchin exclusion treatment and the relative controls (Figure 2). Sea urchins were removed from the upper sides (2.2 m side length) of the treatment plots every 2 or 3 days from July to November 2015 by a free diver, using a metal scraper. In the initial experimental set up, every sampling day, in daylight hours, all the sea urchins from the treatment plots were put into a mesh bag and let then sink over on the nearby bottom. In some instances, the removal method caused sea urchin damage and several fish species, like the wrasses *Coris julis* and *Thalassoma pavo*, the sea breams *Diplodus sargus* and *D*. *vulgaris* together with several species of blennies, fed on the injured sea urchins. Because of their reduced size, these diurnal predators were never observed feeding on uninjured sea urchins (from 2 to 7 cm diameter). As in many breakwaters from the same area where spear and rod fishing are widespread, no large fish predators were ever observed during the study period.

By mid‐November, several observations brought us to suspect that sea urchin grazing was taking place at the treatment plots at night, when no monitoring was performed. It was telling that, in spite of the absence of sea urchins in these plots during daytime, both a distinctive pattern in the epilithic algal matrix (EAM) cover—at the centre of the upper sides of the boulders—and herbivore feeding trails were noticed (Figure 1b).

To assess a possible adjustment to a predominant nocturnal grazing, we decided to conduct a day–night experiment on the same plots. On 11 November 2015, a SCUBA diver conducted five dives, at 10:30 am, 4:30 pm, 10:30 pm, 1:30 am and 8:00 am, recorded the numbers of sea urchins present over the upper sides of the boulders and took photographs of each of them.

In the original experiment, we wanted to measure the effects of sea urchin exclusion on the algae regrowth. We had the same subjects (i.e. 4.84 m^2^ boulder upper surfaces) repeatedly sampled in time (sampling days) for both the exclusion and control treatments. We had the same experimental design for the day–night experiment but, in this case, we wanted to test the effects of exclusion treatment on the abundance of sea urchins. The data from the day–night experiment did not meet some of the basic assumptions for general univariate inference or linear regressions analyses (e.g. a repeated‐measure ANOVA). Most importantly, there was an expected lack of independence between the observations in time (about 6‐h lapse), and the numbers of observations for the day versus night comparisons were unequal (see Quinn & Keough, 2002). Then, in order to estimate the effect of the exclusion treatment (our fixed factor) on the abundance of sea urchins in the day–night experiment, we fitted generalised linear mixed models (GLMM) with either Poisson or negative‐binomial distributions and one or two random factors (see below), and a zero‐inflated Poisson model. Models were run with the packages ‘lm4’ and ‘pscl’ in R 4.2.3. Assumptions were validated and model performance assessed with the ‘performance’ package in R. The best performing model was a GLMM with a negative‐binomial distribution, ‘treatment’ as the fixed factor, ‘time’ (day vs. night) and ‘plots’ as random factors. The same model but without ‘time’ as a random factor was fitted to the daytime (three levels) and the night‐time (two levels) data, separately.

## 3 RESULTS AND DISCUSSION

At the start of the general experiment, in July 2015, the boulder‐exposed surfaces had 6.75 ± 1.75 (mean and SE) individuals of *A. lixula*. The abundance of *P. lividus* was 0.95 ± 0.27 indiv. (mean and SE). This species, which is regularly harvested in the study area by commercial fishermen, was playing a marginal role in grazing on FA at the study site and it was not further investigated in this study.

By the end of August 2015, the same surfaces from the treatment plots sampled during daylight hours were devoid of sea urchins. By this time, we started to observe clumps of sea urchins at the most sheltered junctions between boulders, a common anti‐predator behaviour shown by different sea urchin species (Flukes et al., 2012). In September, an EAM covered different portions of the upper sides of the treatment boulders (see Figure 1c). With the exception of the negligible presence of the red alga *Laurencia* sp. (see Figure 1c), FA were never observed in the sampling plots during the course of the experiment.

**FIGURE 3:**
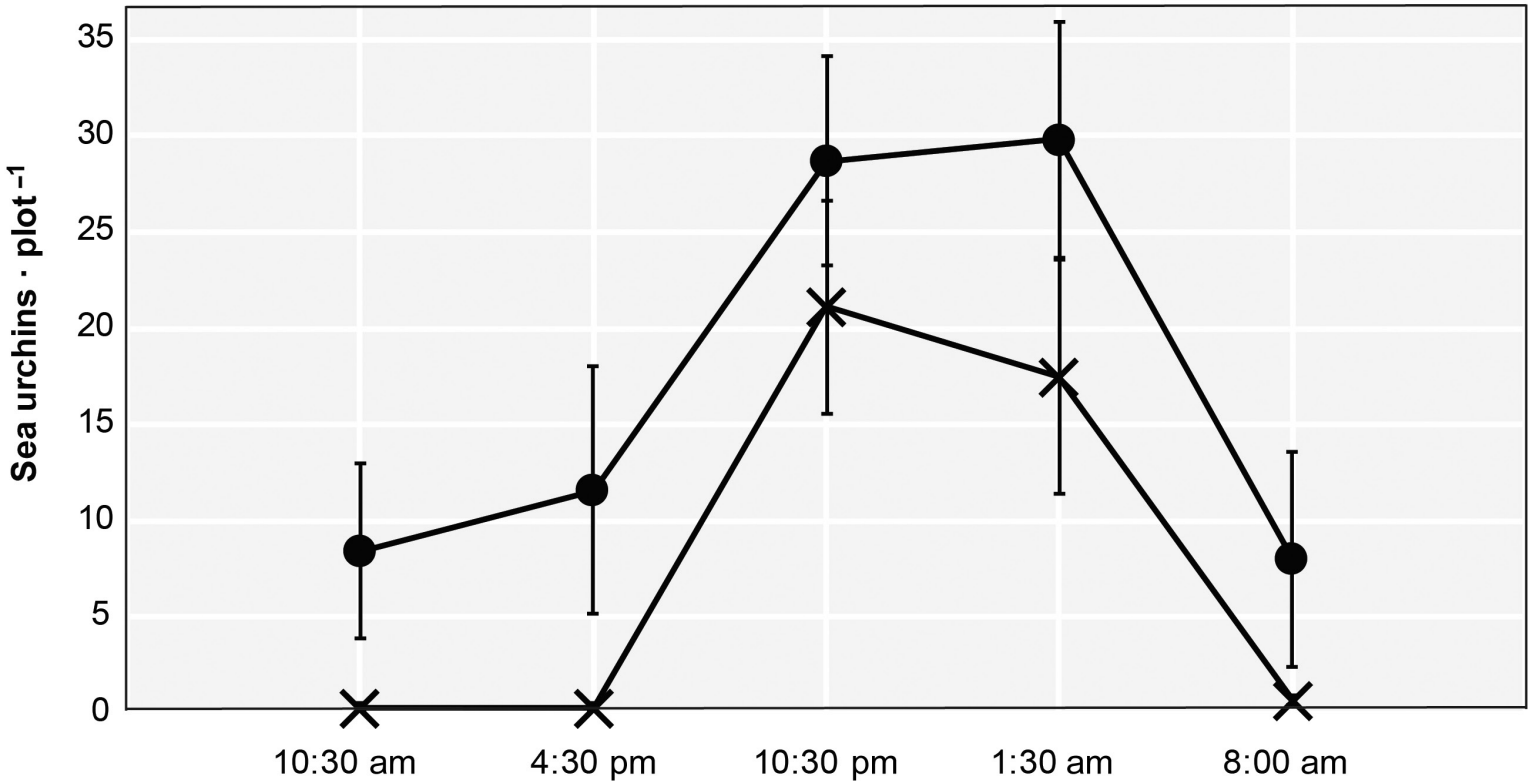
Time course of the abundance of *Arbacia lixula* during the day–night experiment. Mean and standard errors are shown for the treatments (**×**) and the controls (•).

**FIGURE 4:**
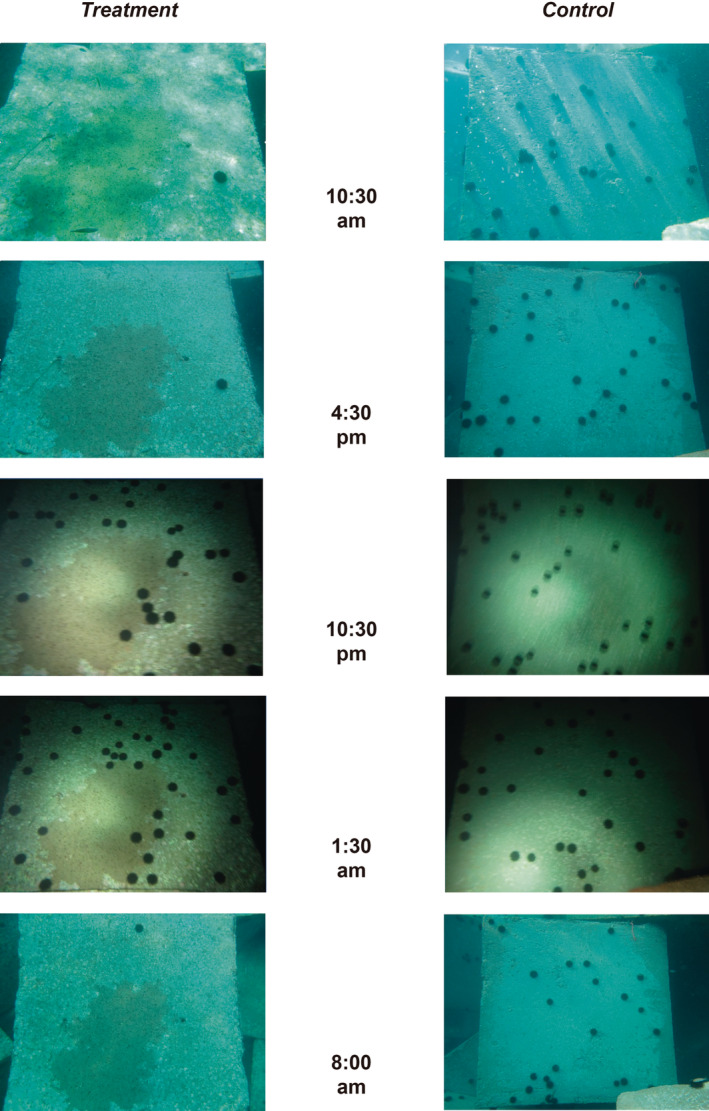
Temporal succession images from a treatment plot (left) and a control plot (right) in the day–night experiment.

We found very strong statistical evidence for a negative effect of the experimental removal of sea urchins on their resulting abundance in the day–night experiment (Table 1a). Notably, when the model was fitted to daylight and night‐time data separately, the evidence for this effect was respectively stronger and weaker than that found when day and night data were pooled together (Table 1b,c). During daylight hours, in fact, the densities of sea urchins were nearly zero in the exclusion plots while they increased equally in both treatments and controls at night (Figures 3 and 4). If depletion due to the experimental removal were the only reason for the absence of sea urchins in daylight hours, we would have expected only some sea urchins at night in the exclusion plots. On the contrary, some of these plots had zero sea urchins during the day and more than 30 (up to 37) at night (Figure 4). It is not so far‐fetched, then, to envisage some adjustment from the sea urchin behaviour between the start of the experiment and the situation recorded in November.

**TABLE 1:** GLMM results.

	Estimate	SE	*Z* value	*p*
(a) Day vs. Night				
(Intercept)	2.6430	0.8417	3.140	<.001
Treatment exclusion	−1.2952	0.3369	−3.845	<.0001
(b) Day				
(Intercept)	1.7418	0.2911	5.983	<.0001
Treatment exclusion	−2.8379	0.5011	−5.664	<.0001
(c) Night				
(Intercept)	3.2964	0.1488	22.152	<.001
Treatment exclusion	−0.4748	0.2133	−2.227	.026

Our results support the idea of a night movement of *A. lixula* from the hidden (mostly underneath) to the upper surfaces of the boulders and they also show a predominant nocturnal foraging behaviour for *A. lixula*.

It is well known that sea urchins may show different foraging behaviours depending on the presence of predators, dead conspecifics and shelters (Andrew & Byrne, 2001; Belleza et al., 2021; Flukes et al., 2012; Galasso et al., 2015; Kitching & Thain, 1983; Morishita & Barreto, 2011; Pagès et al., 2021). While general movement patterns might have been evolutionarily selected for optimising the search of food while escaping predators (Pagès et al., 2021), their adjustment will be modulated by the conditions that sea urchins experience in time and space.

In spite of the limitation of our short‐time assessment in the day‐night experiment (24 h) and in the absence of finfish large enough to prey on adult sea urchins at the study site, we suggest that the presence of dead conspecifics and small fish predators enhanced by our experimental manipulations may have triggered the behavioural adjustment in the grazing patterns in the population of *A. lixula* studied.

Further research is needed to assess whether our findings can be generalised to the behaviour of *A. lixula* in areas where sea urchins are under strong pressure from diurnal predators. In all these cases, it would be recommended to perform sea urchin density counts at night to avoid misleading assessments of herbivore abundance and grazing pressure.

## AUTHOR CONTRIBUTIONS

**Simone Mariani:** Conceptualization (equal); data curation (equal); formal analysis (equal); methodology (equal); software (equal); supervision (equal); validation (equal); visualization (equal); writing – original draft (equal); writing – review and editing (equal). **Susana Pinedo:** Conceptualization (equal); methodology (equal); validation (equal); writing – review and editing (equal). **Esther Jordana:** Conceptualization (equal); methodology (equal); validation (equal); writing – review and editing (equal). **Maria Elena Cefalì:** Conceptualization (equal); methodology (equal); validation (equal); writing – review and editing (equal). **Xavier Torras:** Conceptualization (equal); methodology (equal); validation (equal); writing – review and editing (equal). **Marina Bagur Bendito:** Methodology (equal); writing – original draft (equal); writing – review and editing (equal). **Jana Verdura:** Methodology (equal); writing – review and editing (equal). **Enric Ballesteros:** Conceptualization (equal); funding acquisition (equal); methodology (equal); project administration (equal); supervision (equal); validation (equal); writing – review and editing (equal).

## CONFLICT OF INTEREST STATEMENT

The authors declare no conflict of interest.

## Data Availability

All data used in this research are deposited in: https://zenodo.org/record/8281145.
